# Expression, purification, and characterization of transmembrane protein homogentisate solanesyltransferase

**DOI:** 10.1007/s00253-024-13094-6

**Published:** 2024-03-07

**Authors:** Han Xiao, Long-Can Mei, Hong-Yan Lin, Zhao Chen, Xin-He Yu, Jun Yang, Qiong Tong, Guang-Fu Yang

**Affiliations:** 1 National Key Laboratory of Green Pesticide, International Joint Research Center for Intelligent Biosensor Technology and Health, Central China Normal University, Wuhan, 430079 People’s Republic of China; 2 National Center for Magnetic Resonance in Wuhan, Key Laboratory of Magnetic Resonance in Biological Systems, State Key Laboratory of Magnetic Resonance and Atomic and Molecular Physics, Wuhan Institute of Physics and Mathematics, Innovation Academy for Precision Measurement Science and Technology, Chinese Academy of Sciences, Wuhan, 430071 People’s Republic of China; 3 Wuhan National Laboratory for Optoelectronics, Huazhong University of Science and Technology, Wuhan, 430074 People’s Republic of China

**Keywords:** Homogentisate solanesyltransferase, Membrane protein, Enzyme activity, Bacterial expression system, Molecular docking

## Abstract

**Abstract:**

Homogentisate solanesyltransferase (HST) is a crucial enzyme in the plastoquinone biosynthetic pathway and has recently emerged as a promising target for herbicides. In this study, we successfully expressed and purified a stable and highly pure form of seven times transmembrane protein *Chlamydomonas reinhardtii* HST (*Cr*HST). The final yield of *Cr*HST protein obtained was 12.2 mg per liter of M9 medium. We evaluated the inhibitory effect on *Cr*HST using Des-Morpholinocarbony Cyclopyrimorate (DMC) and found its *IC*_50_ value to be 3.63 ± 0.53 μM, indicating significant inhibitory potential. Additionally, we investigated the substrate affinity of *Cr*HST with two substrates, determining the *K*_m_ values as 22.76 ± 1.70 μM for FPP and 48.54 ± 3.89 μM for HGA. Through sequence alignment analyses and three-dimensional structure predictions, we identified conserved amino acid residues forming the active cavity in the enzyme. The results from molecular docking and binding energy calculations indicate that DMC has a greater binding affinity with HST compared to HGA. These findings represent substantial progress in understanding *Cr*HST’s properties and potential for herbicide development.

**Key points:**

• *First high-yield transmembrane CrHST protein via E. coli system*

• *Preliminarily identified active cavity composition via activity testing*

• *Determined substrate and inhibitor modes via molecular docking*

**Supplementary Information:**

The online version contains supplementary material available at 10.1007/s00253-024-13094-6.

## Introduction

Homogentisate solanesyltransferase (HST), a member of the UbiA superfamily of intramembrane prenyltransferases, plays a critical role in the synthesis of plant plastoquinone (PQ) (Li 2016; Yang et al. 2017). PQ is indispensable for plants, and its deficiency can lead to albino-like symptoms, making HST a promising target for novel herbicides (Shino et al. 2018). The dominant form of PQ in plants is plastoquinone-9 (PQ-9), which is synthesized through a two-stage pathway. The first stage involves the production of the benzoquinone ring precursor and the isopentyl deblock precursor. The second stage involves the condensation of the ring and side chain, followed by subsequent modifications (Liu and Lu 2016). Specifically, HST participates in the second stage of PQ-9 biosynthesis, catalyzing the production of the PQ-9 precursor known as 2-methyl-6-solanesyl-1,4-benzoquinol (MSBQH) from homogentisate (HGA) and solanesyl diphosphate (SDP/SPP). This enzymatic activity is crucial for PQ-9 synthesis and the proper functioning of plants. Understanding the role of HST in PQ-9 biosynthesis provides valuable insights into the underlying mechanisms governing plant growth and development, while targeting HST presents significant potential for the development of selective herbicides that disrupt PQ-9 production, thus offering effective tools for weed control and crop protection.

Recent research publications have highlighted HST as a potential herbicide target within the PQ-9 biosynthesis pathway (Chao et al. 2014; Hunter et al. 2018; Liu and Lu 2016; Sadre et al. 2006, 2010 ; Shino et al. 2018, 2020 ; Tian et al. 2007). One study focused on cyclopyrimorate, a compound developed by Mitsui Chemical in Japan for weed control in rice fields. Through hydrolysis, DMC was generated from Cyclopyrimorate, exhibiting an *IC*_50_ of 3.93 μM against HST (Shino et al. 2018). However, existing commercial HST herbicides have limited effectiveness, necessitating further investigation of the interaction between inhibitors and HST to expand the herbicidal spectrum and develop more potent HST inhibitors (Umetsu and Shirai 2020).

Plant thylakoids and the chloroplast envelope contain membrane proteins that play vital roles in photosynthesis and transport. While some of these proteins act as carriers and channels for ions, chemicals, and even other proteins, a significant number of them have been found to exhibit low or negligible expression (Tan et al. 2008). Addressing the challenge of low expression of plant transmembrane proteins in the *E. coli* system is crucial for understanding their functions and harnessing their potential. Investigating the mechanisms of action of inhibitors and proteins is pivotal in broadening the herbicidal spectrum of HST inhibitors and developing more effective options. Additionally, understanding the expression patterns and functions of membrane proteins in plant thylakoids and the chloroplast envelope can provide valuable insights into photosynthesis, transport processes, and cellular organization, thus contributing to the development of innovative herbicides and a deeper understanding of the intricate mechanisms governing plant physiology.

One of the challenges in the field of membrane protein expression and purification is the limited abundance of exogenous expression (Jensen et al. 2017). Achieving the requisite amount of protein for experimentation often requires a considerable amount of medium, which is both labor-intensive and material-intensive. Additionally, membrane proteins require detergents to be stable in an aqueous solution, and different membrane proteins exist in different forms in different detergents (Bowie 2001; Linke 2009; le Maire et al. 2000; Prive 2007; Seddon et al. 2004; Tanford and Reynolds 1976). Selecting suitable detergents is a critical step in the membrane protein purification process to obtain high-yield and homogeneous membrane proteins for further purification (Lin and Guidotti 2009; Orwick-Rydmark et al. 2016; Smith 2011). Each membrane protein has different physical properties due to the diversity of proteins, emphasizing the importance of identifying a set of expression and purification procedures appropriate to HST to understand its catalytic mode and inhibitor binding mode.

The gene, vector, and expression host are three crucial components in the exogenous expression of proteins (Lin and Guidotti 2009). Their collaboration can increase the yield and quality of the protein. *E. coli*, yeast cells, and insect cells are popular hosts for membrane protein expression (Kesidis et al. 2020), with *E. coli* being the most widely used due to its facile genome manipulation, rapid ploidy, low cost, and broad applicability (Baneyx 1999; Lin and Guidotti 2009; Wagner et al. 2007). Different *E. coli* strains, such as BL21(DE3), BL21(DE3) pLysS, C41(DE3), C43(DE3), BL21-CodonPlus (DE3), Rosetta(DE3), and Lemo21(DE3), are routinely employed to express membrane proteins (Gopal and Kumar 2013; Lin and Guidotti 2009; Rosano et al. 2019; Wiener 2004). For example, a strain screen for seven membrane proteins revealed that while they could not be expressed in strain BL21(DE3), they could be expressed with C41(DE3) and C43(DE3), with C43(DE3) showing significantly better expression (Miroux and Walker 1996). Therefore, testing different vector-expression host combinations is necessary to generate highly expressed proteins.

**Fig. 1:**
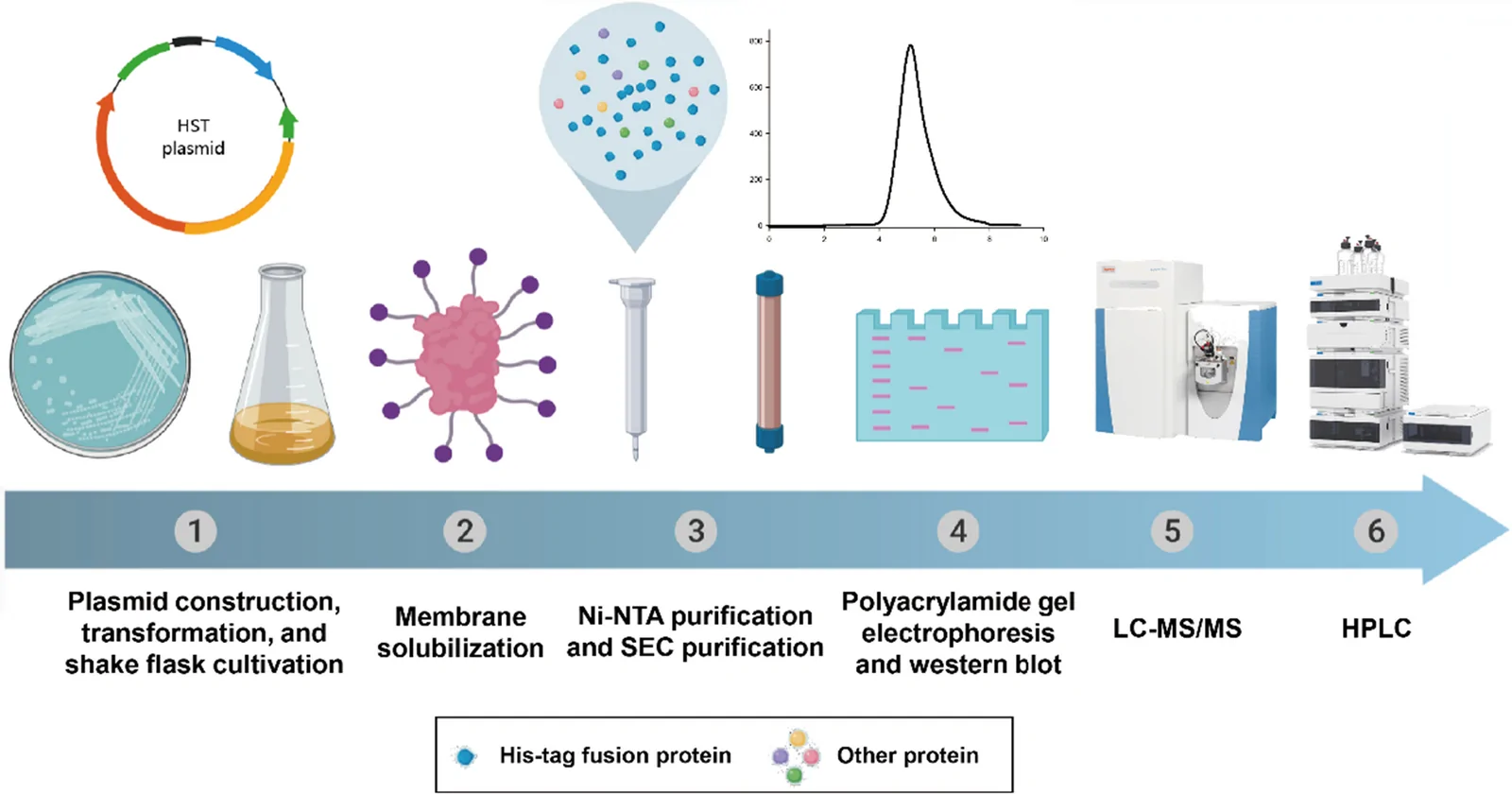
The experimental procedure for HST expression, purification, and characterization (created with MedPeer (www.medpeer.cn))

To obtain HST protein with high yield and purity, this study focused on optimizing the expression and purification of *Cr*HST in the *E. coli* system. The catalytic activity of HST and the inhibitory effect of inhibitors were further measured using the protein with ideal properties. Additionally, we built a three-dimensional structure model of HST using AlphaFold2 to analyze the key residues for catalysis, along with mutagenesis analysis. This work provides a powerful contribution to the subsequent study of the three-dimensional structure of the transmembrane protein HST Fig. 1).

## Materials and method

### Bacterial strains and chemicals

*Escherichia coli* BL21 AI cells were acquired from Thermo Fisher Scientific (Waltham, MA, USA). Farnesyl diphosphate (FPP) and N-lauroylsarcosine sodium salt (NLS) were purchased from Sigma-Aldrich (St. Louis, MO, USA), while homogentisate (HGA) was obtained from TCI (Shanghai, China). DMC was synthesized as described previously (Ikishima 2010).

For detergent screening, we used N-dodecyl-β-D-maltopyranoside (DDM), N-dodecyl phosphocholine (DPC), n-decyl-*N,N*-dimethylglycine (DDGly), n-nonyl-β-D-maltopyranoside (NM), n-dodecyl-*N,N*-dimethylamine-N-oxide (LDAO), glyco-diosgenin (GDN), and lauryl maltose neopentyl glycol (LMNG), all purchased from Anatrace (Maumee, OH, USA).

For purification, we used Ni^2+^-NTA resins from QIAGEN (Duesseldorf, Germany) and Superdex 200 10/300 GL column from GE Healthcare (Little Chalfont, Buckinghamshire, UK).

### Plasmid construction

**Fig. 2:**
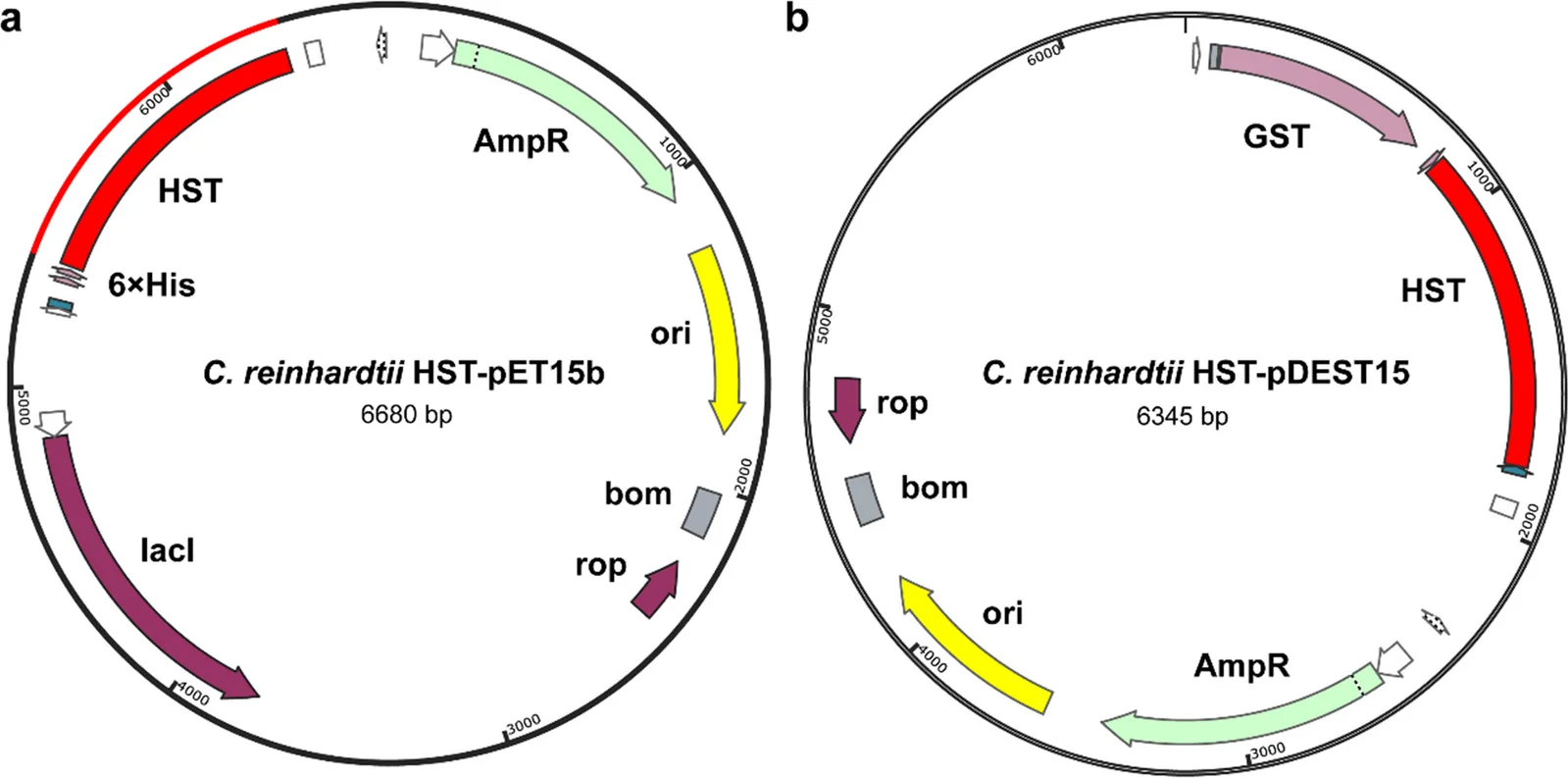
Construction of *Cr*HST into vectors. **a** Construction of *Cr*HST into pET15b vector. **b** Construction of *Cr*HST into pDEST15 vector

The peptide sequences of the *Cr*HST (UniProt code #A1JHN0) lacking putative chloroplast targeting sequences were codon-optimized (supplementary data Fig. S1) and cloned into the pET15b vector with 6 × His-tag followed by a stop codon at an N-terminal of the gene (Fig. 2a). Meanwhile, the same peptide sequences were cloned into the pDEST15 vector with an N-terminal glutathione S-transferase (GST) tag (Fig. 2b).

### HST protein expression

To achieve the overexpression of HST, freshly transformed competent cells containing recombinant pET15b plasmids were cultured in 100 mL LB medium supplemented with 100 μg/mL ampicillin. The cultures were then incubated overnight at 37 ℃ with continuous shaking at 220 rpm. Subsequently, 10 mL of these cell cultures were inoculated into flasks containing either 500 mL of M9 media or 1 L of LB media, both supplemented with 100 μg/mL ampicillin. The cultures were allowed to grow until the cell density reached OD600 = 0.8–0.9. After reaching this point, the cell cultures were further incubated with shaking at 37 °C for a specified period or at 18–20 °C for an extended period before induction.

To induce protein expression, we added 1 mM IPTG to the cell cultures and continued shaking them at 220 rpm for either 5 h at 37 °C or 18–20 h at 18 °C. This induction encouraged the bacteria to express the recombinant protein. Following induction, the cell culture was harvested by centrifugation at 6000 rpm and 4 ℃ for 10 min.

### Optimization of HST protein purification

The cell pellets were suspended in 40 mL of lysis buffer (15 mM Tris, pH 7.5, 150 mM NaCl, 10% glycerol) and sonicated on an ice bath until they became transparent. All subsequent steps were carried out at 4 °C. The cell lysate was then subjected to centrifugation at 6000 rpm for 10 min to obtain the membrane fraction. This membrane fraction was further centrifuged at 20,000 rpm for 1 h, and the resulting supernatant was frozen at –20 °C.

To solubilize the isolated cell membranes, we used 1% (w/v) N-lauroylsarcosine sodium salt or 1% (w/v) N-dodecyl-β-D-maltopyranoside, and the solubilization was performed overnight. The supernatants from the solubilization step were subjected to further centrifugation at 20,000 rpm for 30 min and then treated with nickel-NTA affinity resin for 2 h. The resin was washed in two phases, first with 10 mM and then with 30 mM imidazole in a buffer containing different detergents. The protein was subsequently eluted and concentrated to 0.5 mL using a 30 kDa cutoff Amicon Ultra-15. Size exclusion chromatography (SEC) is a purification method employed to separate substances according to their molecular size. Further purification was carried out using size-exclusion chromatography with a Superdex 200 column in a 20 mM Tris–HCl (pH 7.5) buffer containing different detergents, including DDM, LDAO, NM, GDN, LMNG, DDGly, DPC, and 100 mM NaCl. The peaks were collected and identified through 15% SDS-PAGE.

For protein concentration determination, we measured the absorbance at 280 nm using extinction coefficients of 51,005 M-1 cm^−1^, which were calculated from the amino acid sequences using the ExpASy-ProtParam tool.

### Protein identification by Western blot and LC–MS/MS analysis

The western blot analysis was performed through the general method (Egger and Bienz 1994).

The HST protein was analyzed using 15% SDS-PAGE to separate its components. The separated protein gels were then transferred onto a PVDF membrane. To prevent nonspecific binding, the membrane was blocked using a blocking buffer composed of 5% bovine serum albumin (BSA) in Tris-buffered saline (TBS) containing 2% tween-20. Next, we used an anti-6 × His tag antibody, diluted at a ratio of 1:2000 with the blocking buffer, to probe the membrane and specifically detect the tagged HST protein. The incubation with the primary antibody was carried out at 4 ℃ overnight. After the incubation period, the membrane was washed to remove any unbound antibodies and then incubated with a secondary antibody, goat anti-mouse horseradish peroxidase (HRP) conjugated. Finally, we detected the chemiluminescent signal on the membrane by adding an enhanced chemiluminescence (ECL) substrate, allowing us to visualize and analyze the presence of the tagged HST protein.

The LC–MS/MS analysis was carried out by modifying previously published techniques (Hynek et al. 2006; Wessels et al. 2009). In summary, the sample was dried in a fume hood to remove excess acetonitrile and buffer after being treated with 50% acetonitrile in 40 mM NH_4_HCO_3_ to fade the sample. Next, the sample underwent reduction with 20 mM dithiothreitol at 55 °C for 1 h. It was then alkylated in the dark with 20 mM iodoacetamide in NH_4_HCO_3_ buffer for 30 min. After the alkylation reaction, the sample was washed three times with a 10 mM NH_4_HCO_3_ solution. To achieve an enzyme-to-protein mass ratio of 1:100 in 40 mM NH_4_HCO_3_, the filter was treated with proteinase K. The peptides were then extracted with Oasis HLB cartridges and desalted. The desalting was performed using Oasis HLB cartridges and extraction with 60% acetonitrile/5% formic acid in ddH_2_O.

For separation, C18 AQ beads (3 m, 120, Dr. Masch GmbH, Germany) were placed into the separation column. The mobile phases used were 5% acetonitrile/0.1% formic acid/H_2_O (mobile phase A) and 90% ACN/0.1% FA/10% H_2_O (mobile phase B). A gradient elution mode was employed for better separation, with the following gradient settings: 0–25 min, 3–15% B; 25–40 min, 15–25% B; 40–50 min, 25–40% B; and 50–60 min, 40–100% B.

### Sequence analysis

Isoelectric point and molecular weight were predicted by ProtParam. Multiple sequence alignment was conducted by Multialin (supplementary data Fig. S2) (http://multalin.toulouse.inra.fr/multalin/multalin.html) and optimized by ESPript 3.0 program (https://espript.ibcp.fr/ESPript/cgi-bin/ESPript.cgi). The original codon sequence was aligned with the optimized codon sequence using DNAMAN version 9.0.1.116. The three-dimensional structure was modeled by AlphaFold2 and visualized by PyMOL.

### Enzyme activity assay

For the activity assay, diverging from the purification methods, we continued to follow the testing methods outlined in the literature (Shino et al. 2018, 2020). *E. coli* BL21 AI carrying the pDEST15-HST plasmid was freshly transformed and cultured in 100 mL LB containing 100 μg/mL ampicillin at 37 °C with shaking at 220 rpm for 5 h. Subsequently, 40 mL of the cell cultures were transferred to a flask containing 2 L of LB media with 100 μg/mL ampicillin and further incubated at 37 °C with shaking until the OD_600_ reached 0.4, which took around 2–3 h. To induce the expression of the recombinant protein, 0.2% L-arabinose was added to the culture, and the bacteria were shaken at 220 rpm at 37 °C for 2 h. The cell culture was then harvested by centrifugation at 5000 rpm for 10 min at 4 °C.

**Fig. 3:**
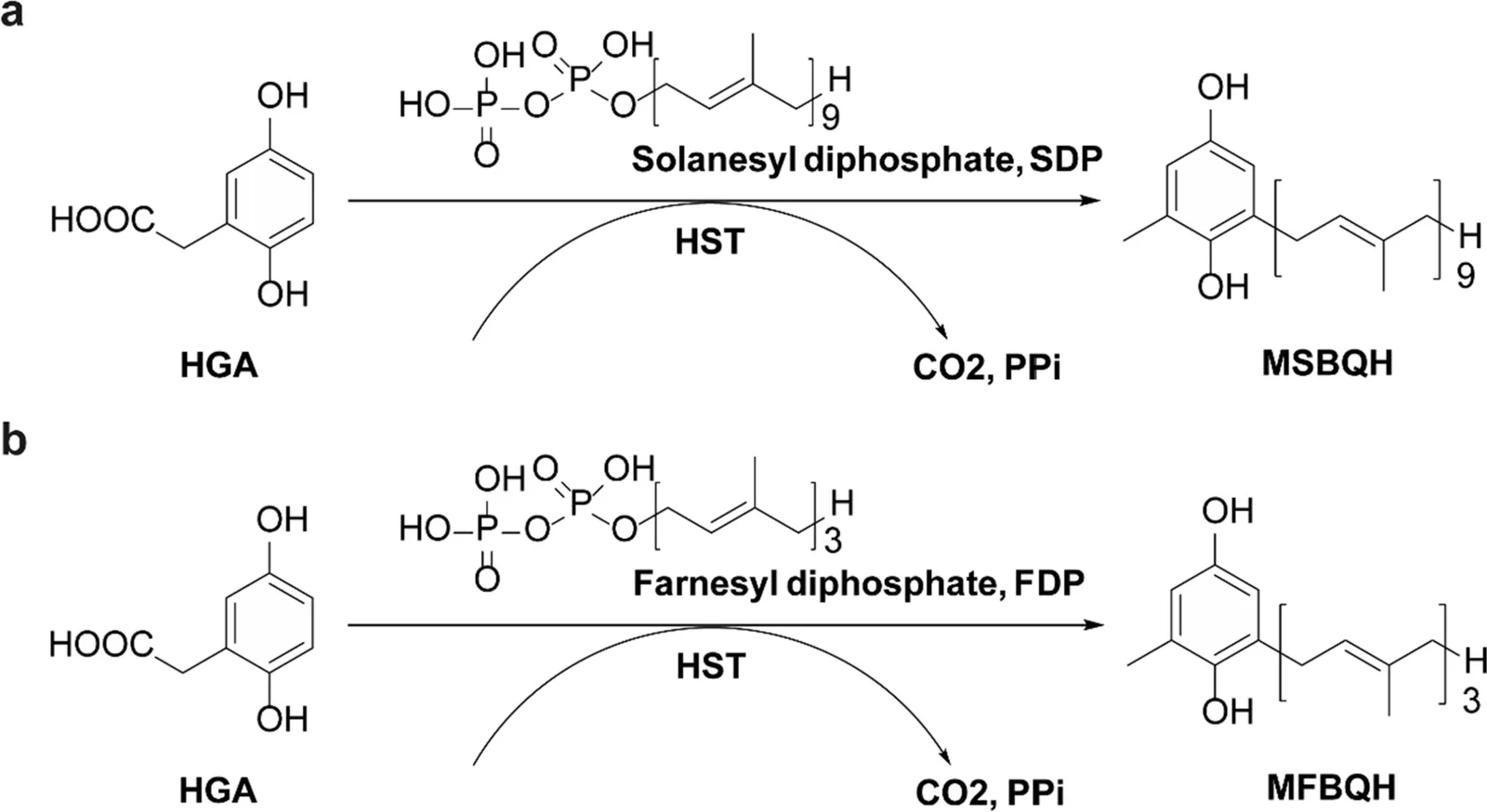
Scheme of HST catalysis. **a** Reaction with solanesyl diphosphate as a substrate. **b** Reaction with farnesyl diphosphate as a substrate

An enzyme activity assay was performed as described previously (Shino et al. 2018, 2020). In this study, the activity of HST was determined by measuring the product MFBQH instead of MSBQH (Fig. 3a and b). For the in vitro HST experiments, a reaction mixture of 100 μL was prepared, containing 50 mM Tricine-NaOH buffer (pH 8.0), 20 mM magnesium chloride, 1.5 mg/mL protein suspension, and various concentrations of substrates, including HGA, farnesyl diphosphate (FPP), a substrate replacing SPP, and DMC. The reaction mixture was incubated at 32 °C for 30 min. To examine the kinetic characteristics of HGA with respect to FPP, the HGA concentration was varied from 0 to 500 μM, while keeping the FPP concentration constant at 100 μM. On the other hand, the FPP concentration was varied from 0 to 100 μM, while maintaining the HGA concentration at 250 μM. After the incubation, each sample received 100 μL of 0.05% sodium borohydride in ethanol, 4 μL of 0.1 M formic acid, and 2.5 μL of 1 M ascorbic acid. Following centrifugation for 10 min at 17,360 × g, the supernatant was analyzed using HPLC. The column was kept at a constant temperature of 30 °C, and the mobile phase was composed of acetonitrile and 0.1% formic acid in a 65:35 (v/v) ratio. The effluent was detected using a fluorescence detector (Ex. 290 nm, Em. 330 nm), with a flow rate of 1.0 mL/min.

### Molecular docking

The stereo conformations of the compound HGA and DMC were established by SYBYL software. Since no crystal structure of *Cr*HST is available, we downloaded the AlphaFold2 predicted structure from AlphaFold Protein Structure Database. The AutoDock 4.2 program (Morris et al. 2009) was used to dock the compounds into the substrate binding site (residues N135, D139, K147, K207, D269, and D273). The Lamarkian genetic algorithm was applied for the docking conformational search. The grid size was set to 60 × 60 × 60 Å, and the grid space was set to 0.375 Å. The grid center was set to make the grid box cover the whole catalytic site. The other parameters were set as default values. Among a set of 100 candidates of dock poses, the best candidate was selected according to the standard AutoDock scoring function and the binding mode of the catalytic site.

### Binding-free energies calculated by MM/GBSA

MM/GBSA method (Wang et al. 2019) was applied to evaluate the binding free energies of HST with the two compounds. Molecular dynamics simulation of the protein-compound complex was performed by AMBER 16 software (Case et al. 2005). The protein molecule was parameterized with AMBER ff14SB force field (Maier et al. 2015), and the ligand molecule was parameterized with the general AMBER force field (gaff) (Wang et al. 2004). The complex system was solvated with TIP3P water model (Price and Brooks 2004) with a distance of 10 Å between the solute and box. Counter-ions (Na^+^ and Cl^−^) were added to neutralize the solvated system. The solvated system was then minimized. After minimizations, the system was gradually heated from 0 to 300 K in the NVT (T = 300 K) ensemble over a period of 500 ps and then relaxed in the NPT ensemble (T = 300 K and P = 1 atm). All the covalent bonds involving hydrogen atoms were constrained by the SHAKE algorithm (Yoneya et al. 1994). The cut-off value of 10 Å was set to calculate the short-range interactions (electrostatic and Van der Waals interactions). Particle Mesh Ewald (PME) algorithm (Darden et al. 1993) was applied to calculate the long-range electrostatic interactions. The time step was set to 2 fs, and the snapshots were recorded every 10 ps. A 5 ns production simulation is performed. Finally, 100 structural snapshots were extracted for binding free energy calculations. The binding-free energy for a protein-compound complex was calculated according to the standard protocol of MM/GBSA implemented in AMBER software.

## Results

### *C. reinhardtii* HST expression, purification, and identification

**Fig. 4:**
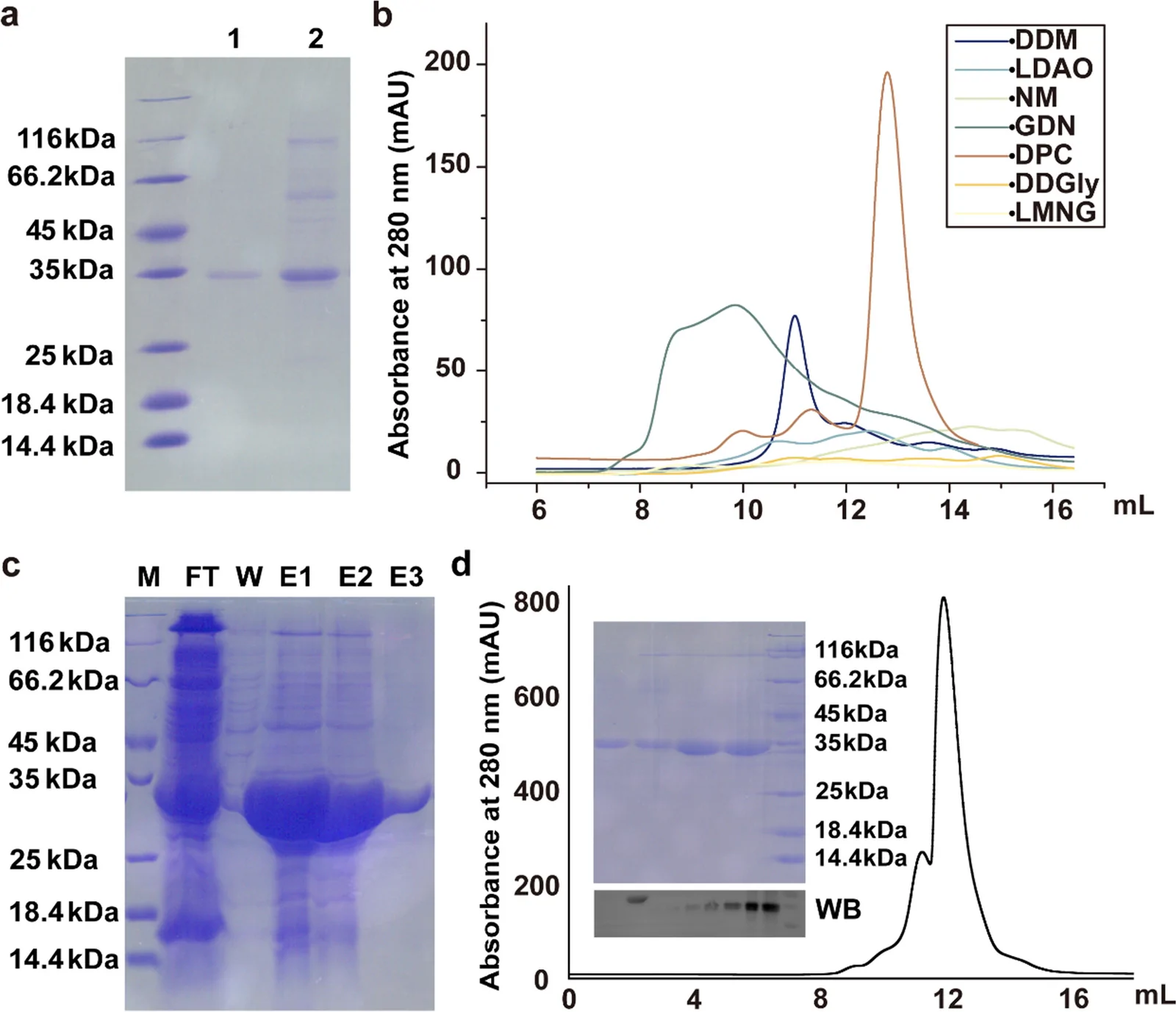
Purification of *Cr*HST protein. **a** SDS-PAGE of *Cr*HST using DPC and DDM to purify. Lane1 *Cr*HST in DPC and lane 2 *Cr*HST in DDM. **b** Homogeneity of *Cr*HST in different detergents analyzed by gel filtration.** c** *Cr*HST was purified with the Ni^2+^-NTA column. SDS-PAGE determination from chromatograms refers to M, marker; FT, flow-through; W, washed with 10 mM imidazole; and E, elution with 300 mM imidazole. **d** *Cr*HST was purified with a size-exclusion chromatography column. SDS-PAGE showed elution peaks of purified protein. Western blot for protein identification

To obtain the *Cr*HST protein, we conducted separate expressions in LB and M9 media, followed by membrane solubilization using either DDM or NLS. The purification process involved affinity chromatography, and the target protein was eluted from the nickel-NTA resin using DDM or DPC buffer (Fig. 4a). We observed that when LB medium was used, the obtained protein was not a pure *Cr*HST protein; it seemed to form a complex (supplementary data Fig. S3). However, the purified protein containing only *Cr*HST was successfully obtained when bacteria were cultured in M9 medium. Therefore, to achieve a high yield of single *Cr*HST protein, we explored expressing HST proteins in different species of competent cells and culturing at different temperatures in M9 medium (supplementary data Fig. S4). Among the four different expression strains tested, we found that protein yields were higher at 18 °C compared to 37 °C. Rosetta 2(DE3) displayed the best expression at 18 °C, with a protein yield of 12.2 mg per liter of media (Table 1).

**Table 1:** Yield of *Cr*HST in different competent cells at different temperatures

Competent cell	BL21(DE3)		Rosetta 2(DE3)		C43(DE3)		BL21-CodonPlus (DE3)	
Temperature (℃)	18	37	18	37	18	37	18	37
Total protein (mg per liter)	6.5	4.4	12.2	9.6	1.8	2.6	6.1	4.6
Protein yield (mg/bacterial dry weight/g)	17.6 (18 ℃)		23.5 (18 ℃)		1.5 (18 ℃)		5.8 (18 ℃)	

Detergent screening is a critical step in purifying membrane proteins. We tested three types of detergents: non-ionic, ionic, and amphoteric. Specifically, we used DDGly (ionic detergent), NM, DDM, GDN, and LMNG (non-ionic detergents), as well as LDAO and DPC (amphoteric detergents). Different detergents can affect proteins in different ways. Consequently, we conducted detergent screening for *Cr*HST and found that *Cr*HST did not homogeneously aggregate in all detergents except DPC and DDM (Fig. 4b). As previously mentioned, *Cr*HST formed a complex in DDM. Therefore, we transformed the pET15b-HST expression vector into competent cell Rosetta 2(DE3), expressed it in M9 medium, solubilized the membranes with NLS, and then purified the protein using Ni^2+^-NTA chromatography (Fig. 4c) and gel filtration chromatography with DPC, resulting in high homogeneity HST protein with over 95% purity (Fig. 4d). Protein yield was determined using the BCA method.

To verify the expression of the recombinant HST proteins, we performed western blotting, detecting the 6 × His-tag at the N-terminus of the recombinant with anti-His tag antibody. Furthermore, LC–MS/MS results confirmed the correct HST sequences with 76.8% sequence coverage (supplementary data Fig. S5).

### Enzyme activity assay

**Fig. 5:**
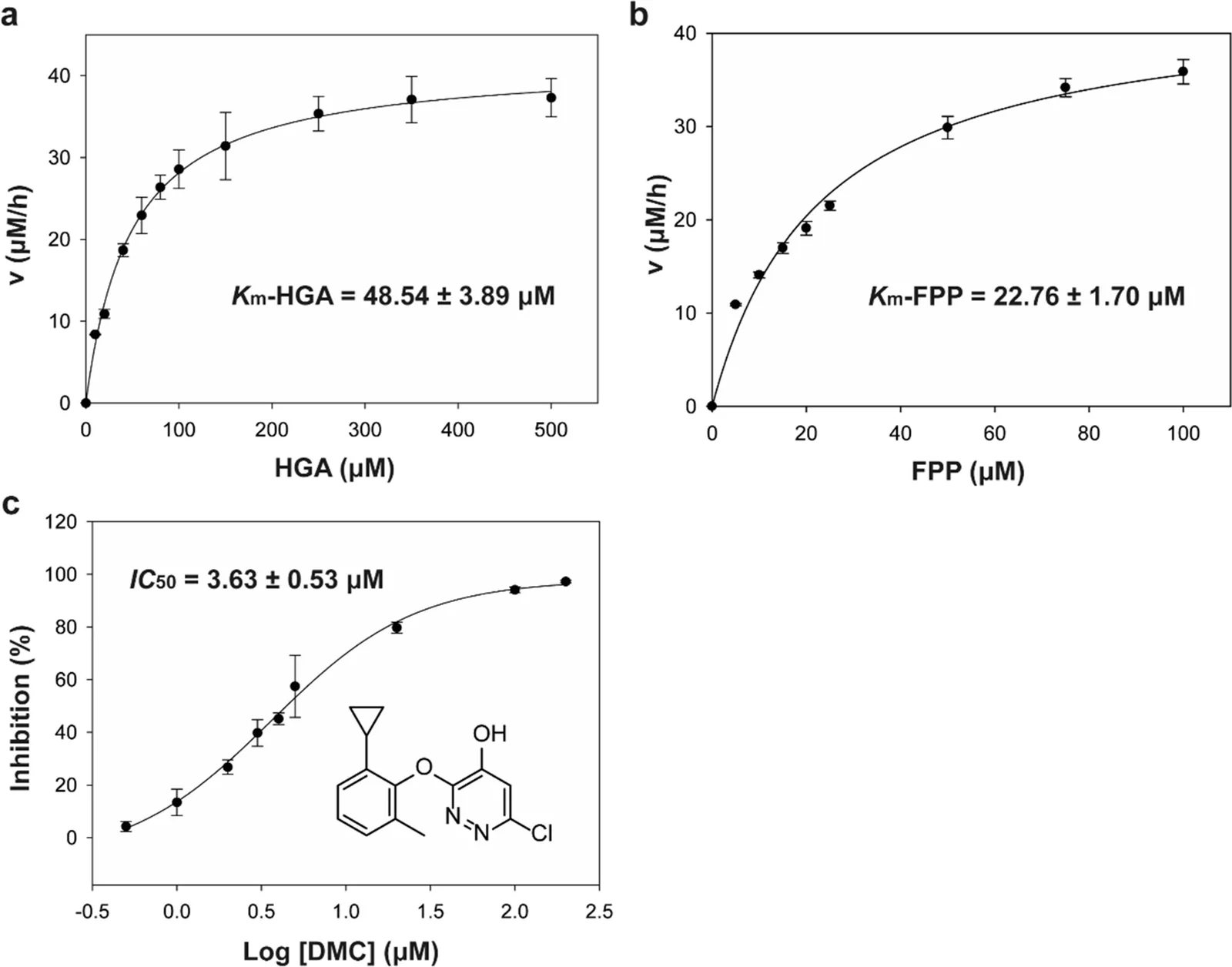
Enzymatic activity assay. **a** Michaelis constant of HGA to *Cr*HST. **b** Michaelis constant of FPP to *Cr*HST. **c** Determination of the *IC*_50_ of HST inhibitor DMC

To investigate the activity and enzymatic characteristics of *Cr*HST, we conducted an assay using the HPLC method to monitor the formation of the product MFBQH. By using HGA and FPP as substrates, we determined the kinetic constants of *Cr*HST. The *K*_m_ value for HGA was found to be 48.54 ± 3.89 μM when the concentration of FPP was fixed at 100 μM (Fig. 5a), while the *K*_m_ value for FPP was determined as 22.76 ± 1.70 μM when the concentration of HGA was fixed at 250 μM (Fig. 5b).

The only commercially available inhibitor of HST was cyclopyrimorate, produced by Mitsui Chemical in Japan, which inhibited HST upon hydrolysis to DMC. To assess the blocking capacity of HST action, we evaluated the HST inhibitor DMC and found it to have an *IC*_50_ value of 3.63 ± 0.53 μM (Fig. 5c). A summary of the Michaelis constants of *Cr*HST and the *IC*_50_ value of DMC can be found in Table S1.

### Sequence analysis and identification of critical catalytic residues

**Fig. 6:**
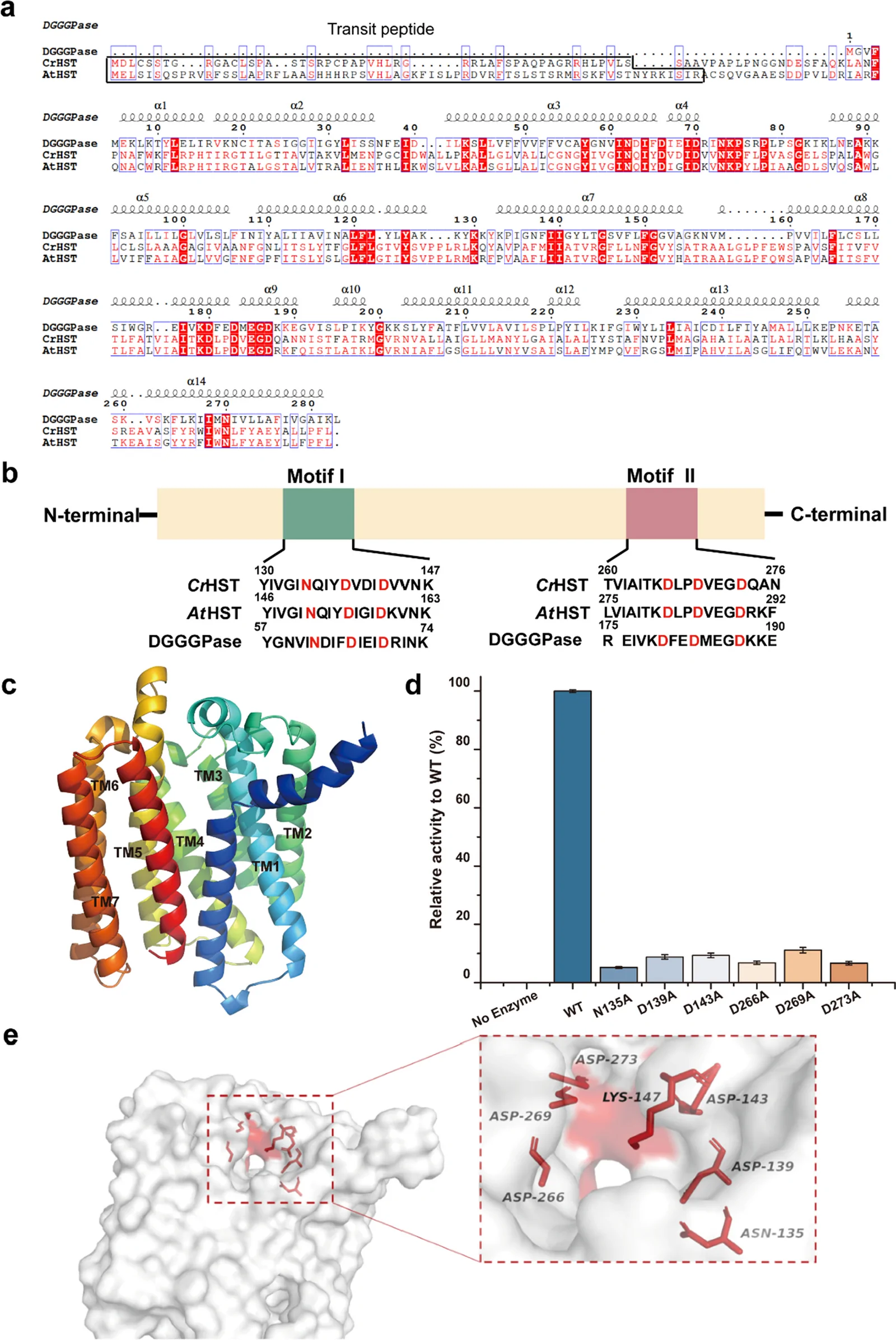
Sequence analysis of HST and activity assay. **a** *Cr*HST, *At*HST and DGGGPase sequence alignment. **b** Alignment of conserved sequence motifs. The key residues of two Asp-rich motifs are highlighted in red. **c** *Cr*HST is a transmembrane protein with 7 predicted transmembrane helices. The transmembrane domain is labeled. **d** The enzyme activity assay of *Cr*HST mutants. **e** The putative activity pocket of *Cr*HST

The HST gene was successfully cloned from cDNA in *C. reinhardtii*, and its open reading frame (ORF) encoded 370 amino acids. Uniprot prediction revealed the presence of a 50 amino acid signal peptide within *Cr*HST. As an atomic resolution three-dimensional structure of HST had not been reported, we utilized the Swiss-model for homology modeling and identified the three-dimensional structure of DGGGPase, which showed the highest similarity to HST (Fig. 6a). However, upon conducting sequence analysis, we found that the primary structure similarity between *Cr*HST and DGGGPase was only 20%. Nonetheless, we identified two conserved aspartic acid motifs, I and II, in various species using sequence alignment data and by analyzing the catalytically active cavity of other UbiA superfamily members (Fig. 6b) (Brauer et al. 2004, 2008; Cheng and Li 2014; Harish et al. 2013). Notably, *Cr*HST is a seven-time transmembrane protein (Fig. 6c). By comparing it with the three-dimensional structure of HST predicted by AlphaFold2, we gained theoretical guidance in identifying the active cavity of HST. Consequently, we focused on targeted mutations and activity tests on the conserved amino acid residues.

To evaluate the significance of the two aspartate motifs on catalytic activity, we introduced mutations in the conserved residues within the N_135_XXXD_139_XXXD_143_ and D_266_XXD_269_XXXD_273_ motifs, including Asn135, Asp139, Asp143, Asp266, Asp269, and Asp273. The results of the catalytic activities of these mutants are displayed in Fig. 6d. The experimental findings demonstrated that when these two aspartate motifs were changed to alanine, the catalytic activity of HST diminished, suggesting their involvement in the catalytic process of HST.

Subsequently, we approximated the composition of the catalytically active cavity. The AlphaFold2 prediction tool proved to be a powerful tool for predicting three-dimensional structures in recent years. Utilizing the *Cr*HST structure model predicted by AlphaFold2, we identified the corresponding aspartate motifs and found that they were predominantly clustered in the upper opening of the barrel protein (Fig. 6e).

### Molecular docking and binding free energies calculation

**Fig. 7:**
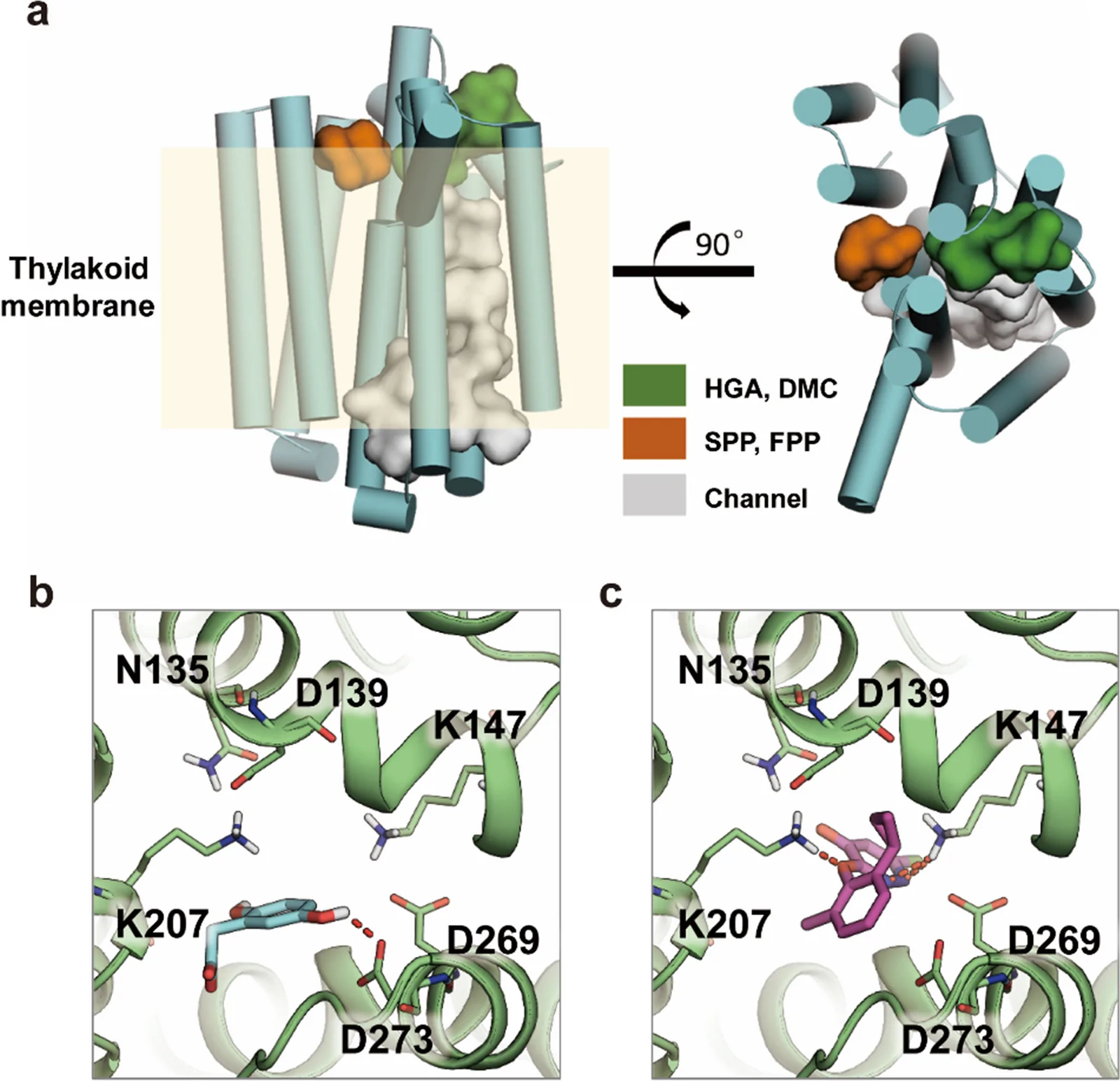
Proposed binding modes of *Cr*HST with substrate and inhibitor. **a** Possible binding pocket of *Cr*HST. **b** Proposed binding modes of *Cr*HST with HGA. **c** Proposed binding modes of *Cr*HST with DMC. *Cr*HST protein was shown as green cartoon, and the interacting residues were highlighted as stick style. The compounds HGA and DMC were shown as cyan and magenta sticks, respectively

In our study, we identified potential binding sites on the *Cr*HST protein structure, as depicted in Fig. 7a. Specifically, two binding cavities (colored green and orange) were discovered on the external surface of *Cr*HST’s thylakoid membrane. Notably, the green cavity aligns with observed mutations, leading us to speculate that it could potentially serve as a binding site for HGA or DMC. Conversely, the orange cavity could potentially bind to substrates like FPP and SPP. A distinct white cavity forms a transmembrane channel, composed of *Cr*HST's helical bundles.

Furthermore, we visualized the simulated binding interactions of *Cr*HST with both HGA and DMC in Fig. 7b and c. Specifically, HGA forms a hydrogen bond with residue D273, while DMC establishes three hydrogen bonds with residues K147 and K207. Notably, DMC resides deeper within the binding pocket compared to HGA.

In addition, we quantified the binding strengths of these compounds using MM-GBSA methods (see Table 2). The results revealed binding free energies of -15.39 kcal/mol for HGA and -18.40 kcal/mol for DMC, respectively. This suggests that DMC exhibits a stronger binding affinity with *Cr*HST than HGA.

**Table 2:** Binding-free energies (kcal/mol) of HST with HGA and DMC calculated by MM/GBSA

Compound	ΔE_ELE_	ΔE_VDW_	ΔE_GBSUR_	ΔE_GB_	ΔG
HGA	-19.50	-13.64	-2.19	19.94	-15.39
DMC	-38.22	-19.35	-3.85	-43.02	-18.40

## Discussion

In this study, we successfully achieved the production and isolation of the *Cr*HST protein, which represents a novel herbicide target (Shino et al. 2018, 2020). The refined protein yielded 12.2 mg with a purity of over 95%, demonstrating the efficacy of our purification approach in obtaining a stable and pure HST protein within the bacterial expression system. The obtained *K*_m_ values for FPP and HGA (*K*_m_-FPP = 22.76 ± 1.70 μM, *K*_m_-HGA = 48.54 ± 3.89 μM) were consistent with previous reports in the literature (Sadre et al. 2006, 2010). Furthermore, by aligning homologous protein sequences (Ren et al. 2020), we identified some conserved amino acid residues, and our enzyme activity assay led us to identify the crucial catalytic residues, namely Asn135, Asp139, Asp143, Asp266, Asp269, and Asp273. These amino acids play pivotal roles within the active cavity, actively participating in the catalytic reaction of HST. Meanwhile, molecular docking shows HGA forms a hydrogen bond with D273, while DMC forms three hydrogen bonds with K147 and K207, deeper in the pocket. Using MM-GBSA, we calculated binding strengths: -15.39 kcal/mol for HGA and -18.40 kcal/mol for DMC, suggesting stronger affinity of DMC with *Cr*HST than HGA. This achievement lays a solid foundation for future investigations into the three-dimensional structure of HST, a crucial step in understanding its function and interactions.

The large-scale expression of heterologous functional membrane proteins in *E. coli* remains a challenging task, and our research is confronted with this challenge as well. The first challenge in this endeavor is to achieve efficient expression of stable membrane proteins. In our study, we identified two crucial factors for the success of our work: the selection of bacterial strains and the induction temperature. We also screened the expression of HST proteins from different species and observed that for various HST proteins, employing Rosetta2(DE3) and inducing expression at a lower temperature (18 °C) resulted in higher protein yields (data not shown). However, the conditions for the high-level expression of different membrane proteins in *E. coli* are specific, and there is no universally applicable condition for achieving high expression of every membrane protein. Overall, optimizing bacterial strains, expression temperature, induction OD value, and additives, as well as employing various protein truncation forms, may still yield relatively favorable results. Nevertheless, there are still numerous membrane proteins that require expression in eukaryotic systems to be effectively expressed.

The second challenge we encountered in our work is obtaining the target protein with activity. Despite the successful purification, we encountered some challenges in fully characterizing the enzymatic catalytic function of the purified HST protein. To ensure the rapid and successful establishment of the in vivo assay system, we adopted the protein expression vector and induction system previously employed in the literature (Shino et al. 2018, 2020), namely, the use of pDEST15 and BL21 AI competent cells. However, in the subsequent purification process, structural studies may be conducted through crystallization or solid-state nuclear magnetic resonance (NMR). The oversized GST tag on pDEST15 can affect protein crystallization, and the introduction of GST tags can hinder the application of solid-state NMR structure analysis methods, particularly for low molecular weight proteins. Therefore, in the expression and purification experiments, the tag was replaced with a 6 × His tag, which has a smaller molecular weight and virtually no impact on any experiments. For activity assay, in the initially extracted membrane fractions, we detected the activity of the protein catalyzing substrate reactions. However, the protein did not exhibit catalytic activity in various detergents, while weak catalytic activity was observed in samples that the protein was reconstructed into liposomes. These experimental results suggest, firstly, that detergents may potentially impact the protein conformation, leading to protein inactivation. Alternatively, the encapsulation by detergents may prevent the exposure of the active site, making it inaccessible to substrates. This is supported by the observation that proteins in crude membrane fractions, not subjected to detergent extraction, exhibit normal activity. Secondly, detergents or phospholipids may occupy the catalytic center pocket of the HST enzyme. The substrate SPP or FPP of HST contains a phosphate group, similar to the phosphate groups present in phospholipids. Considering these factors, obtaining stably active HST proteins in the presence of detergents or lipids poses a key challenge for our subsequent work.

In addition to the aforementioned challenges, another focal point of this study is the observation that HST exists in different forms depending on the culture medium and detergent used. Throughout the research, we found that HST can only be expressed and purified as an individual protein using M9 medium and DPC detergent. When employing conventional LB medium and the widely used DDM detergent, the obtained protein tends to be in a complex form. One possible explanation for this phenomenon is the impact of detergents on the stability of the membrane protein complex. This complex appears to be more stable in DDM but less stable in DPC. Another potential explanation is related to the LB medium, which comprises naturally derived components with inherent complexity, including unidentified constituents. It remains uncertain whether these unknown components may contribute to the stability of the protein complex. In contrast, the M9 medium has a simpler composition, consisting solely of salts, carbon sources, and nitrogen sources.

Nevertheless, our research does have certain limitations. Despite achieving high purity and homogeneity of the target protein, obtaining the three-dimensional structure of a transmembrane protein like HST remains extremely challenging. We recognize that cryo-electron microscopy, X-ray diffraction, and solid-state NMR are potential approaches for elucidating the structure (Loquet et al. 2013; Venien-Bryan et al. 2017). Further investigations are necessary to determine which method is most suitable for HST and overcome the obstacles in determining its three-dimensional structure. Our current expression, purification, and characterization of critical catalytic residues, however, provide a strong basis for future structural studies. In conclusion, our successful expression and purification of the HST protein contribute significantly to enzyme inhibitor design, structural biology, and investigations into protein catalytic mechanisms. Our findings lay the groundwork for further advancements in understanding HST's function and its potential applications as a target for herbicides. We have proposed a set of methods for the expression and purification of multi-transmembrane proteins. The exploration of such multi-transmembrane proteins holds significant implications for other transmembrane proteins, potentially offering new research avenues for understanding and studying other transmembrane proteins.

## Supplementary Information

Below is the link to the electronic supplementary material.

Supplementary file1 (PDF 540 KB)

## Data Availability

All data generated and analyzed in this study are included in this article or the supplementary information. Plasmids encoding the enzyme constructs studied in this work are available upon reasonable request.
