# Supplementary material for: Expression, purification, and characterization of transmembrane protein homogentisate solanesyltransferase
Source: Appl Microbiol Biotechnol. 2024 Mar 7;108(1):256. doi: 10.1007/s00253-024-13094-6 (PMC10920428; doi:10.1007/s00253-024-13094-6)
Supplement: Supplementary file 1 — Supplementary file1 (PDF 540 KB) [file 253_2024_13094_MOESM1_ESM.pdf]

Journal name:

Applied Microbiology and Biotechnology

Title:

Expression, purification, and characterization of transmembrane protein Homogentisate solanesyltransferase

Han Xiao,[1] Long-Can Mei,[1] Hong-Yan Lin,[1] Zhao Chen,[1] Xin-He Yu,[1] Jun Yang,[2,3] Qiong Tong,[2,3, *] Guang-Fu Yang[1, *]

1 National Key Laboratory of Green Pesticide, International Joint Research Center for Intelligent Biosensor Technology and Health, Central China Normal University, Wuhan 430079, P.R. China

2 National Center for Magnetic Resonance in Wuhan, Key Laboratory of Magnetic Resonance in Biological Systems, State Key Laboratory of Magnetic Resonance and

Atomic and Molecular Physics, Wuhan Institute of Physics and Mathematics, Innovation Academy for Precision Measurement Science and Technology, Chinese Academy of Sciences, Wuhan 430071, PR China

3 Wuhan National Laboratory for Optoelectronics, Huazhong University of Science and Technology, Wuhan 430074, PR China

***Corresponding author:** Qiong Tong, tongqiong@wipm.ac.cn; Guang-Fu Yang, gfyang@mail.ccnu.edu.cn

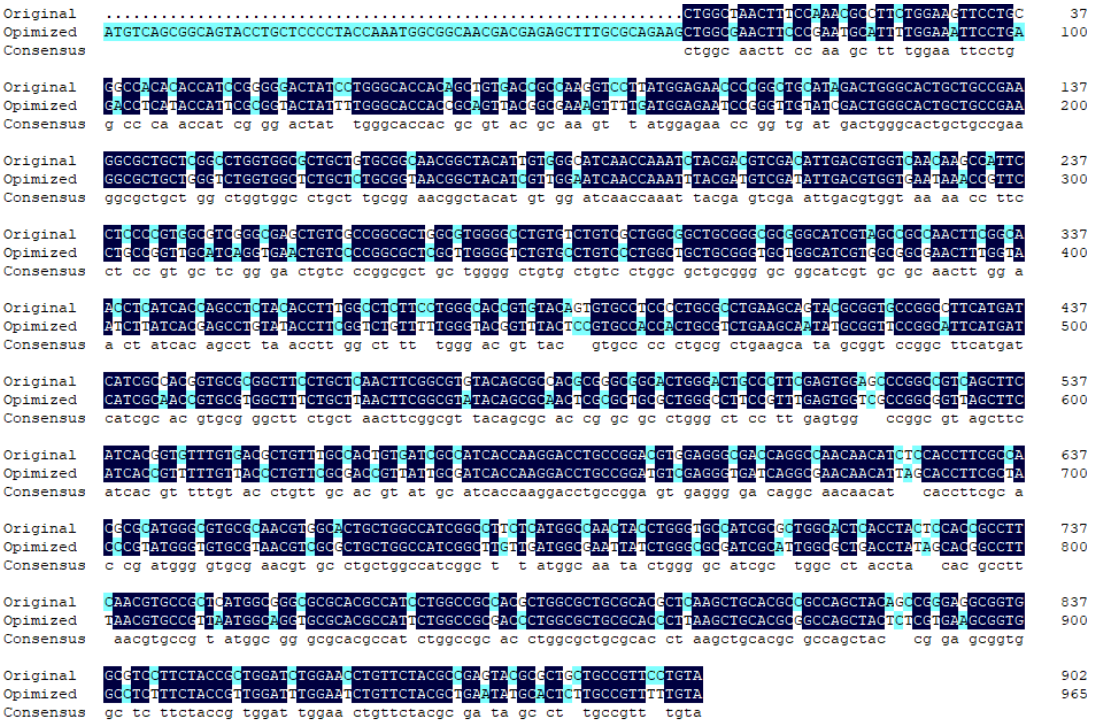

Figure S1. Comparative of original codons and optimized codons of *Cr*HST.

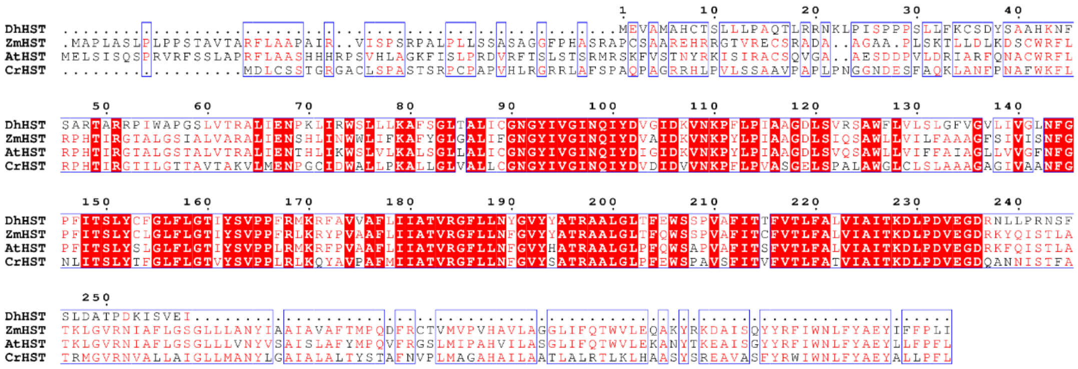

Figure S2. Sequence alignment of different species of HST.

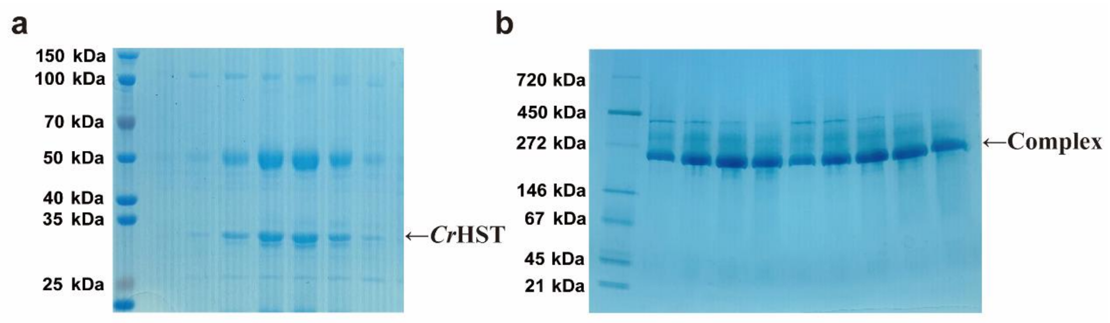

Figure S3. Purification of *Cr*HST complex protein in LB medium. **a** SDS-PAGE of *Cr*HST complex. **b** BN-PAGE of *Cr*HST complex.

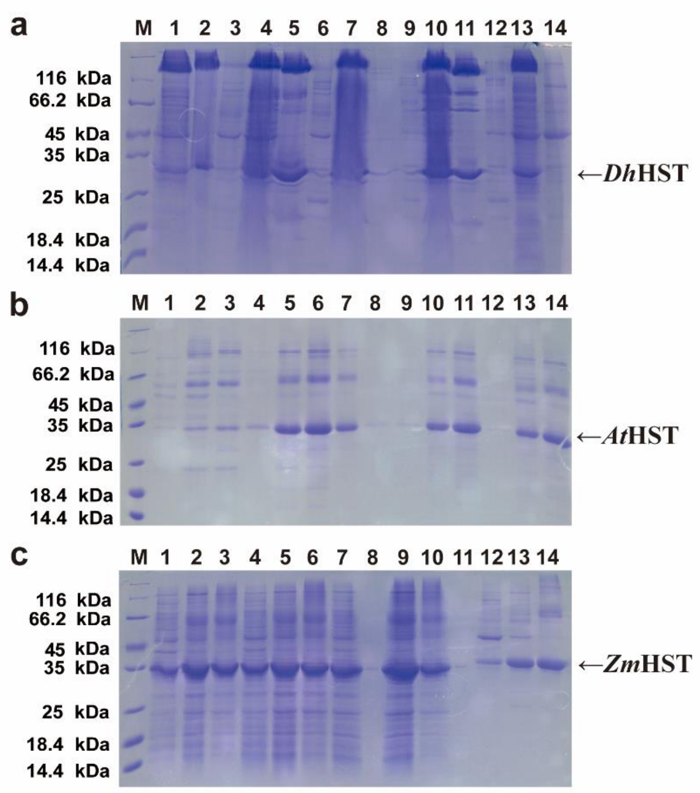

Figure S4. SDS-PAGE results of purified HST proteins of different species. **a** Ni^{2+}-NTA purification of *Dorcoceras hygrometricum* HST (*Dh*HST). **b** Ni^{2+}-NTA purification of *Arabidopsis thaliana* HST (*At*HST). **c** Ni^{2+}-NTA purification of *Zea mays* HST (*Zm*HST).

MHHHHHHSAA VPAPLPNGGN DESFAQKLAN FPNAFWKFLR PHTIRGTILG TTAVTAKVLM

ENPGCIDWAL LPKALLGLVA LLCGNGYIVG INQIYDVDID VVNKPFLPVA SGELSPALAW

GLCLSLAAAG AGIVAANFGN LITSLYTFGL FLGTVYSVPP LRLKQYAVPA FMIIATVRGF

LLNFGVYSAT RAALGLPFEW SPAVSFITVF VTLFATVIAI TKDLPDVEGD QANNISTFAT

RMGVRNVALL AIGLLMANYL GAIALALTYS TAFNVPLMAG AHAILAATLA LRTLKLHAAS

YSREAVASFY RWIWNLFYAE YALLPFL

Figure S5. The amino acid sequence of the *Cr*HST. LC-MS/MS analysis found the amino acid sequence of HST with 76.8% sequence coverage. The matched peptides are shown in red.

Table S1. Kinetic parameters of *Cr*HST

Michaelis constants assay			Half-maximal inhibitory concentration (IC_{50}) assay	
Substrate	Counter-substrate	K_m (μM)	Compound	IC_{50} (μM)
FPP	250 μM HGA	22.76 ± 1.70	DMC	3.63 ± 0.53
HGA	100 μM FPP	48.54 ± 3.89		
